# Willingness to pay for footwear, and associated factors related to podoconiosis in northern Ethiopia

**DOI:** 10.1093/inthealth/ihw033

**Published:** 2016-09-27

**Authors:** Girmay Tsegay, Abreham Tamiru, Tsige Amberbir, Gail Davey, Kebede Deribe

**Affiliations:** a School of Public Health, College of Medicine and Health Sciences, Debre Markos University, P.O. Box 269, Debre Markos, Ethiopia; b National Podoconiosis Action Network, Addis Ababa, Ethiopia; c International Orthodox Christian Charities, Addis Ababa, Ethiopia; d Wellcome Trust Brighton & Sussex Centre for Global Health Research, Brighton, BN1 9PX, UK; e Schools of Public Health, Addis Ababa University, Addis Ababa, Ethiopia

**Keywords:** Footwear, Neglected tropical disease, Northern Ethiopia, Podoconiosis, Shoes

## Abstract

**Background:**

In Northern Ethiopia, use of footwear by the rural community is limited, and non-governmental organizations provide footwear for school children as a means of preventing podoconiosis. However, this is not a sustainable strategy. This study assessed willingness to pay for footwear among people with and without podoconiosis.

**Methods:**

A comparative cross-sectional community-based study was conducted in Mecha and Gozamen woredas among randomly selected people with and without podoconiosis. Trained health extension workers collected data using an interviewer-administered structured questionnaire. The data were entered into EPI-Data and exported to SPSS version 16.0 statistical software package for analysis.

**Results:**

The willingness to pay for footwear among people with and without podoconiosis was 72.3% and 76.7% respectively (p=0.30). People with podoconiosis in the lower quintiles of economic status were less likely to be willing to pay for footwear than those in the higher quintiles.

**Conclusions:**

There is substantial willingness to pay for footwear. The expressed willingness to pay indicates demand for footwear in the community, suggesting an opportunity for shoe companies. There are still a substantial proportion of individuals not willing to pay for footwear. This requires intensified public education and social transformation to bring about change in behavior towards footwear use if elimination of podoconiosis within our generation is to be achieved.

## Introduction

Podoconiosis is a form of elephantiasis that predominantly affects barefoot subsistence farmers in areas of red volcanic soil.^1^ Podoconiosis is characterised by bilateral swelling of the lower legs with mossy and nodular changes to the skin, and causes considerable disability.^2,3^ Although the aetiology is not fully understood, current evidence suggests genetic susceptibility and the role of mineral particles from irritant volcanic soils.^3^ Podoconiosis follows a chronic course causing progressively increasing disability with continued exposure to irritant soils.^4,5^ Early stage disease can easily be treated by foot hygiene, bandaging and footwear.^4^ Nonetheless, podoconiosis interventions are only found in a few endemic areas, and the disease is poorly understood by many of Ethiopia's healthcare professionals.^6^

Podoconiosis prevention strategies include use of footwear, regular foot hygiene practice and covering house floors.^7^ Evidence shows that for certain neglected tropical diseases (NTD), namely Buruli ulcer, cutaneous larva migrans, tungiasis, any soil transmitted helminth infection, strongyloidiasis and leptospirosis, using footwear is associated with reduced odds of infection.^8,9^

In Northern Ethiopia use of footwear by the rural community is limited, and non-governmental organizations like International Orthodox Christian Charities (IOCC) provide footwear for school children as a means of preventing podoconiosis.^10,11^ Given the widespread practice of walking barefoot, addressing the need for footwear is challenging. Free distribution of shoes may appear to be an immediate solution, but its sustainability is questionable. A relatively small proportion of the school age children who would benefit from shoes have been included in the free footwear distributions so far. To have a lasting impact and high coverage, it is important to introduce cost recovery mechanisms.^10^ Cost recovery will have potential benefits: first, individuals who have paid some money may utilize footwear better because of a sense of ownership. Second, the money collected can be used to distribute more footwear to others in need. Third, once the demand is created, it should be possible to engage private organizations to take over such activities.^12–14^

Willingness to pay for a certain product or service can be assessed using three criteria: judgment about probable benefit from the service (relative to other needs, desire and capacity given the price and the cost of the service), clients’ needs and desires, and information about the existence of the service. Willingness to pay for a certain product or service can be assessed using two different methods: the contingent evaluation method and choice experiments.^15–17^

Studying the willingness to pay for a certain product or service enables government and non-governmental organizations to make decisions regarding user fees for a range of services.^18^ The Ethiopian government has adopted self-financing and promoted user fees for certain services like purchase of drugs, supplies, and salary supplements.^19^ People of higher socio-economic status are more likely to pay for a service or product. Charging these people will help to extend the service or product to poorer people. A study conducted among patients in northern Ethiopia measured willingness to pay for ‘podoconiosis treatment services’, of which shoes are one component. A total of 72.8% of participants in this study were willing to pay for podoconiosis treatment services. Half of the study participants (45% of women and 54.5% of men) wore shoes during the interview, and 135 study participants (34.4%; 38.1% of women and 31.8% of men) said that they had never worn shoes.^10^ Three-quarters of participants were willing to pay at least 50% of shoe production costs, if other treatment services were provided free of charge.^10^ This study was focused on willingness to pay for shoes as part of a treatment package, but this is distinct from willingness to pay for footwear as a means of prevention. The current study was conducted in a podoconiosis-endemic district to assess the willingness to pay for footwear among people with and without podoconiosis. This will be important for potential service expansion, and public-private partnership for increasing access to shoes to prevent podoconiosis.

## Methods

### Study area

Amhara region is the second most populous region in Ethiopia, with a total population of 19 272 508 people. The region has 11 administrative zones, including East and West Gojam zones, and 170 districts (woredas). Mecha woreda (located in West Gojam) and Gozamen woreda (in East Gojam) were included in this study.^20^

### Study design and sampling

A comparative cross-sectional community-based study was conducted in Mecha and Gozamen woredas among randomly selected adults with and without podoconiosis.

First we purposively selected East and West Gojam zones from Amhara region, then using a lottery method, we randomly selected Mecha woreda from West Gojam and Gozamen woreda from East Gojam. Since the disease is more common in rural than urban areas, we randomly selected four rural kebeles from each woreda.^4^ Participants with podoconiosis had already been identified and registered by health extension workers for the purpose of treatment in IOCC treatment centres, but for the purpose of this study, the health extension workers were re-trained to ensure correct identification. People with podoconiosis aged 18 years and above, who had lived for more than 6 months in the study area, were selected from the registration lists using a random sampling method. People without podoconiosis were selected from the nearest neighbours of people with podoconiosis, were also aged 18 years and above and had lived more than 6 months in the study area.

A total sample size of 468 (234 people with podoconiosis and 234 without) was determined using a general formula for two population proportions by taking the level of significance to be 5% and the power 90%. In a recent study on willingness to pay for podoconiosis lymphoedema treatment, the proportion willing to pay was 72.8%.^21^ Since footwear is one component of treatment, we used this proportion and an expert-based difference of 15% between people with and without podoconiosis. We arrived at this 15% difference by discussion with podoconiosis experts and researchers. A non-response rate of 5% was also added.

### Data collection tool and procedures

Data were collected using a structured questionnaire. The tool was adapted from the previous study conducted in the same region.^10^ This questionnaire was translated into the local language (Amharic) and then back-translated into English by a third party to ensure accuracy. We used colour photos to help participants identify podoconiosis. Questions on socio-demographic and socio-economic characteristics, footwear practice, perceptions of podoconiosis, and willingness to pay for footwear were included. For leather shoes the cost was estimated at 250 Ethiopian Birr (ETB) (US$11.50) and for canvas shoes, 150 ETB (US$7), based on market prices.

To assess willingness to pay for footwear, the contingent valuation method (CVM) was used. CVM is a questionnaire-based method used to elicit the monetary value a person is willing to pay for a health care service.^15,17^ The CVM includes constructing a hypothetical market for the commodities and asking individuals the maximum amount they would be willing to pay for the service or the minimum amount they would be willing to accept in compensation if they were deprived of it. Techniques used in contingent evaluation are binary, with follow-up, where a price for the commodity is presented to the respondent and they are asked to give a ‘yes’ or ‘no’ response.

Eight health extension workers were hired to collect data in each woreda (district). In each kebele (the smallest administrative unit of the Ethiopian government), one nurse was assigned to supervise them. To maintain the quality of data, data collectors and supervisors were trained by the principal investigator for three days. Topics covered during the training included objectives of the study, interviewing techniques and practical exercises. Pilot testing of the questionnaire was done in a neighboring village before the actual data collection. Data were collected in February 2015.

### Data analysis

The collected data were cleaned, coded, entered into EPI-Data version 3 (The EpiData Association, Odense, Denmark) and transferred and analysed using SPSS computer software, version 16 (IBM, Armonk, NY, USA). Socio-demographic summary statistics were presented using frequencies, tables and graphs. Bivariate analysis was done and variables with p-value <0.20 were included in the multiple logistic regression analysis, which was performed to assess the association between willingness to pay for footwear and various explanatory variables. A p-value of <0.05 was taken as the cut-off for significance in multiple logistic regression. Odds ratios and 95% confidence intervals were also computed along with corresponding p-values. We used principal component analysis to generate a wealth index using 16 possible assets: electricity, radio, clock, television, mobile, refrigerator, separate room used for kitchen, separate place for cattle, bicycle, farmland, cattle, savings/bank account, type of floor, type of roof, type of wall and toilet. We used quintiles to categorize the wealth index based on the Ethiopian Demographic and Health Survey (EDHS) 2011.^22^

### Ethical considerations

Ethical approval was obtained from Amhara Regional Health Bureau. A letter of support was obtained from East and West Gojam zone health offices. Informed oral consent was obtained from every study participant, and recorded by an independent witness.

## Results

### Socio-demographic characteristics of study participants

A total of 420 individuals (188 people with podoconiosis and 232 people without podoconiosis) participated in the study, giving a response rate of 90%. Most (295, 70.2%) respondents were male. Only one-fifth (34, 18.1%) of people with podoconiosis and 52 (22.4%) of people without podoconiosis were able to read and write, respectively. Most people with podoconiosis (141, 75.0%) and without podoconiosis (199, 85.8%) were married. Almost all participants were of Amhara ethnicity. The majority (253, 60.1%) of the study participants had two to three family members and 380 (90.5%) of the study participants were farmers.

The mean (SD) age of people with and without podoconiosis was 45±13 and 42±10 years old, respectively, and the mean (SD) number of years lived in their current areas was 38.5±17 and 36±13 years respectively (Table 1).

**Table 1.** Socio-demographic characteristics of people with and without podoconiosis in northern Ethiopia, February 2015

Variables	With podoconiosis (n=188)	Without podoconiosis (n=232)
	n (%)	n (%)
Age		
18–34	45 (23.9)	49 (21.1)
35–40	42 (22.3)	66 (28.4)
41–50	37 (19.7)	74 (31.9)
51+	64 (34)	43 (18.5)
Original place of residence		
Rural	185 (98.4)	228 (98.3)
Urban	3 (1.6)	4 (1.7)
Sex		
Male	124 (66)	171 (73.7)
Female	64 (34)	61 (26.3)
Occupation		
Farmer	172 (91.5)	208 (89.7)
Others	14 (8.5)	24 (10.3)
Marital status		
Married	141 (75)	199 (85.8)
Single	20 (10.6)	14 (6)
Separated/Divorced	15 (8)	5 (2.2)
Widowed	12 (6.4)	14 (6)
Educational status		
Can read and write	34 (18.1)	52 (22.4)
Cannot read and write	154 (81.9)	180 (77.6)
Years lived in the area (quartiles)		
3–26	50 (26.6)	48 (20.7)
27–36	40 (21.3)	66 (28.4)
37–46	34 (18.1)	74 (31.9)
47+	64 (34)	44 (19)
Number of female family members		
One	50 (26.6)	58 (25)
Two to three	112 (59.6)	140 (60.3)
Four and more	26 (13.8)	34 (14.7)
Number of male family members		
One	58 (30.9)	71 (30.6)
Two to three	93 (49.5)	118 (50.9)
Four and more	26 (19.7)	43 (18.5)

### Socio-economic characteristics of study participants

Most study participants (131, 72.9% with podoconiosis and 171, 73.7% without) perceived their household income to be ‘average’ in reference to their neighbours. More people without podoconiosis were found in the highest wealth index quintile and more people with podoconiosis in the lowest (Table 2).

**Table 2.** Economic characteristics of people with and without podoconiosis in East and West Gojam Zone, February, 2015

Variables	With podoconiosis (n=188)	Without podoconiosis (n=232)
	n (%)	n (%)
Perceived household income		
Below average	50 (26.6)	44 (19)
Average	137 (72.9)	171 (73.7)
Above average	1 (0.5)	17 (7.3)
Average monthly income		
<500 ETB (US$23)	57 (30.3)	64 (27.6)
501–800 ETB (US$23–36.90)	54 (28.7)	41 (17.7)
801–1500 ETB (US$37–69)	38 (20.2)	74 (31.9)
>1501 ETB (US$69)	39 (20.7)	53 (22.8)
Wealth index quintile		
Highest	16 (8.5)	65 (28)
Fourth	46 (24.5)	41 (17.7)
Middle	45 (23.9)	50 (21.6)
Second	39 (20.7)	34 (14.7)
Lowest	42 (22.3)	42 (18.1)

ETB: Ethiopian Birr.

### Shoe wearing practices of study participants

A pair of shoes had been owned by 134 (71.3%) of people with podoconiosis and 150 (64.7%) of people without at some point in their life. Two-thirds (126, 67.0%) of people with podoconiosis owned a pair of shoes at the time of data collection, compared to 142 (61.2%) of people without. Most people with podoconiosis stated that they wore shoes to go to market and church (174, 92.6% and 149, 79.3%, respectively). Most of the study participants wore shoes during special events like funerals, village meetings and weddings. However, few wore shoes while working inside the house, fetching water or harvesting.

Almost half (88, 47.0%) of people with podoconiosis and 111 (48.0%) without were not wearing any type of footwear during the interview. Reasons given for this included not being able to afford footwear (106, 56.5% of people with podoconiosis and 120, 51.6% of people without), and not seeing the benefit of footwear (38, 20.3% of people with podoconiosis and 31, 13.2% of people without). A significant number (24, 13.0%) of people with podoconiosis and 28 (12.1%) of people without reported that they could not find the correct size.

### Knowledge of participants about footwear and podoconiosis

The vast majority (179, 95% of people with podoconiosis and 211, 90.9% of people without) believed that footwear was important. Nearly half (88, 46.8%) of people with podoconiosis and 108 (46.6%) without said they had not heard of podoconiosis. Of those who had heard of it, 58 (58.0%) of people with podoconiosis and 66 (53.2%) of people without believed the cause of the disease to be evil spirits. Only 12 (12.0%) and 14 (11.3%) of people with and without podoconiosis, believed the disease to be caused by the soil. Most (132, 72.0%) of people with and 149 (64.0%) of people without podoconiosis thought that podoconiosis was preventable (Table 3).

**Table 3.** Knowledge about podoconiosis and footwear among people with and without podoconiosis in northern Ethiopia, February 2015

Variables	With podoconiosis (n=188)	Without podoconiosis (n=232)
	n (%)	n (%)
Footwear is important (n=420)		
Yes	179 (95.2)	211 (90.9)
No	9 (4.8)	21 (9.1)
Importance of shoe wearing		
Prevent from disease	171 (96.1)	203 (95.8)
Increase confidence	24 (13.5)	29 (13.7)
Help to work hard	19 (10.7)	28 (13.1)
Other	15 (8.1)	6 (2.8)
Heard of podoconiosis		
Yes	100 (53.2)	124 (53.4)
No	88 (46.8)	108 (46.6)
Cause of podoconiosis (n=224^a^)		
Magic	58 (58)	66 (53)
Cold air	8 (8)	3 (2.4)
Clay soil	12 (12)	14 (11.3)
Hereditary	20 (20)	32 (25.8)
Others (insect, dirty water, snake bite)	2 (2)	9 (7.5)
Podoconiosis is preventable		
Yes	132 (70.2)	149 (64.2)
No	56 (29.8)	83 (35.8)
Method of podoconiosis prevention		
Avoid working during cold air	24 (18.2)	14 (9.4)
Avoid insect bite	1 (0.8)	4 (2.7)
Avoid marriage with a patients	11 (8.3)	19 (12.8)
Use footwear	91 (68.9)	86 (57.7)
Others	5 (3.8)	26 (17.4)
Information from health extension workers		
Yes	61 (32.4)	59 (25.4)
No	127 (67.6)	173 (74.6)
Information from mass media		
Yes	20 (10.6)	44 (19)
No	168 (89.4)	188 (81)

^a^ Those individuals who had heard about podoconiosis: 100 people with podoconiosis and 124 without.

### Perceptions of the study participants about podoconiosis

Almost half the people with podoconiosis 93 (49.5%) but only 14 (6%) of people without podoconiosis reported an affected family member. People without podoconiosis tended to have fewer affected family members (Table 4).

**Table 4.** Risk perception towards podoconiosis among people with and without podoconiosis in northern Ethiopia, February 2015

Variables	With podoconiosis (n=188)	Without podoconiosis (n=232)
	n (%)	n (%)
At risk of getting the disease		
Strongly agree	53 (28.2)	76 (32.8)
Agree	62 (33.0)	80 (34.5)
Strongly disagree	73 (38.8)	76 (32.8)
Action should be taken to prevent podoconiosis		
Strongly agree	29 (15.4)	49 (21.1)
Agree	25 (13.3)	53 (22.8)
Strongly disagree	134 (71.3)	130 (56.0)
Family members affected		
Yes	93 (49.5)	14 (6.0)
No	95 (50.5)	218 (94.0)

### Willingness to pay for footwear

Overall, almost three-quarters 314 (74.8%) of respondents were willing to pay for footwear. Slightly fewer people with podoconiosis than without (72.3% vs 76.7%, p=0.30) were willing to pay for footwear, but this difference was not statistically significant. The median amount that people with and without podoconiosis were willing to pay for footwear was 100 and 120 ETB (approximately US$5 and US$6), respectively. Of those who were willing to pay for footwear, one-quarter (35, 25.7%) of people with podoconiosis and nearly one-third (58, 32.6%) of people without were willing to pay the full cost of leather footwear; the corresponding figures for canvas shoes were 40 (30.7%) and 63 (35.4%). The majority of people with (118, 62.8%) and without (172, 74.1%) podoconiosis were willing to pay for shoes for their families (Table 5).

**Table 5.** Willingness to pay (WTP) for footwear among people with and without podoconiosis in northern Ethiopia, February 2015

Variables	Podoconiosis		
	Yes	No	p-value
	n (%)	n (%)	
WTP for footwear (n=420)			
Yes	136 (72.3)	178 (76.7)	NS
No	52 (27.7)	54 (23.3)	
WTP for leather footwear (n=314)			
WTP full cost (250 ETB) (US$11.50)	35 (25.7)	58 (32.6)	
WTP 90% (225 ETB) (US$10.40)	50 (36.7)	69 (38.8)	NS
WTP 85% (213 ETB) (US$10)	8 (5.8)	20 (11.2)	
WTP for canvas footwear (n=314)			
WTP full cost (150 ETB) (US$7)	40 (30.7)	63 (35.4)	NS
WTP 85% (128 ETB) (US$6)	54 (41.5)	68 (38.2)	
WTP 70% (105 ETB) (US$5)	36 (27.8)	42 (23.5)	
Willingness to pay for their families (n=420)			
Yes	118 (62.8)	172 (74.1)	0.02^a^
No	70 (37.2)	60 (25.9)	

ETB: Ethiopian Birr; NS: not significant; WTP: willingness to pay.

^a^ Significant: p<0.05.

**Figure 1.**
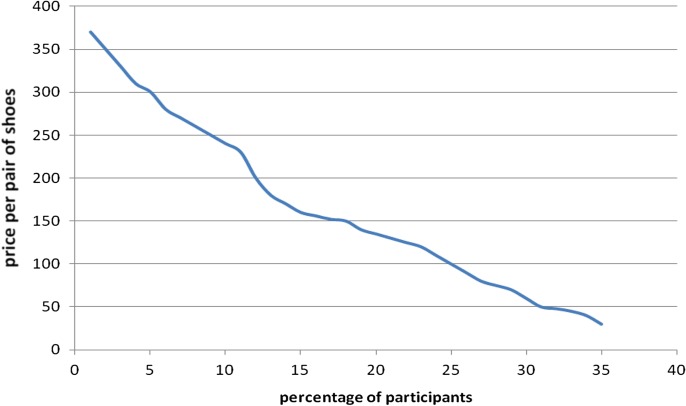
Percentage of participants willing to pay by price at which a pair of shoes is offered among people with and without podoconiosis in northern Ethiopia, February 2015.

Figure 1 shows the percentage of people willing to pay a specified price for a pair of shoes. When asked ‘What is the maximum amount that you would be willing to pay for a pair of shoes?’ only 2% percent of the participants were willing to pay 370 ETB (US$18.50), while around 10% were willing to pay more than 250 ETB (US$12.50) and 30% were willing to pay 50 ETB (US$2.50) or less.

### Factors associated with willingness to pay for footwear among people without podoconiosis

The following variables were entered into the model after binary logistic regression: sex, age, occupation, educational status, marital status, perceived income, ever worn shoes, heard about podoconiosis, heard from media, heard from health extension workers, action can be taken to prevent podoconiosis, number of years lived and wealth index quintile. The multivariate analysis showed that being single, disagreeing that action can be taken to prevent podoconiosis and being in the lowest quintile of the wealth index were all associated with lower willingness to pay among people without podoconiosis. Perceiving one's income to be ‘average’ was associated with greater willingness to pay (Table 6).

**Table 6.** Stepwise multivariate analysis of willingness to pay for footwear among people without podoconiosis in northern Ethiopia, February 2015

Variables	Willingness to pay among people without podoconiosis				
	Yes	No	COR (95% CI)	AOR (95% CI)	p-value
Marital status					
Married	166	33	1	1	
Single	5	9	0.1 (0.04, 0.35)	0.07 (0.02, 0.38)	0.002
Divorced/Separated	3	2	0.3 (0.05, 1.89)	1.3 (0.17, 9.86)	NS
Widowed	4	10	0.08 (0.03, 0.27)	0.2 (0.05, 1.00)	NS
Perceived household income					
Below average	17	27	1	1	
Average	149	22	10.7 (5, 22.8)	4.7 (1.68, 13.29)	0.003
Above average	12	5	3.8 (1.1, 12.7)	1.5 (0.28, 9.86)	NS
Action can be taken to prevent podoconiosis					
Strongly agree	68	8	1	1	
Agree	64	16	0.47 (0.19, 1.17)	0.45 (0.13, 1.6)	NS
Strongly disagree	46	30	0.18 (0.08, 0.43)	0.15 (0.05, 0.50)	0.002
Wealth index quintile					
Highest	60	5	1	1	
Fourth	34	7	0.4 (0.12, 1.4)	0.2 (0.0.05, 1.02)	NS
Middle	43	7	0.5 (0.15, 1.72)	0.42 (0.09, 1.98)	NS
Second	26	8	0.3 (0.08, 0.9)	0.2 (0.05, 1.10)	NS
Lowest	15	27	0.05 (0.02, 0.14)	0.1 (0.03, 0.56)	0.007

AOR: Adjusted odds ratio; COR: crude odds ratio; NS: not significant.

### Factors associated with willingness to pay for footwear among people with podoconiosis

The following variables were entered to the model after binary logistic regression: sex, age, occupation, educational status, marital status, perceived income, ever worn shoes, heard about podoconiosis, heard from media, heard from health extension workers, action can be taken to prevent podoconiosis, number of years lived and wealth index quintile.

Among people with podoconiosis, multivariate analysis showed that being female, being unable to read or write, having never worn footwear, not knowing about podoconiosis, not having had information from health extension workers and being in the lowest wealth index quintile were associated with lower willingness to pay for footwear (Table 7).

**Table 7.** Stepwise multivariate analysis of willingness to pay for footwear among people with podoconiosis in northern Ethiopia, February 2015

Variables	Willingness to pay among people with podoconiosis				
	Yes	No	COR (95% CI)	AOR (95% CI)	p-value
Sex					
Male	100	24	1	1	
Female	36	28	0.3 (0.16, 0,6)	0.4 (0.13,0.67)	0.006
Educational status					
Able to read and write	25	9	1	1	
Unable to read and write	111	43	0.9 (0.4, 2.2)	0.2 (0.05, 0.8)	0.012
Ever used footwear					
Yes	101	33	1	1	
No	35	19	0.6 (0.3, 1.2)	0.3 (0.11, 0.78)	0.001
Heard of podoconiosis					
Yes	86	14	1	1	
No	50	38	0.2 (0.1, 0.4)	0.2 (0.05, 0.64)	0.001
Heard information from health extension workers					
Yes	55	6	1	1	
No	81	46	0.2 (0.07, 0.48)	0.2 (0.05, 0.78)	0.001
Years lived					
3–26 years	40	10	1	1	
27–36 years	27	13	0.6 (0.19, 1.35)	0.16 (0.04, 0.73)	0.001
37–46 years	29	5	1.5 (0.45, 4.69)	2.3 (0.36, 14.3)	
47+ years	40	24	0.4 (0.18, 0.98)	0.08 (0.02, 0.35)	0.004
Action can be taken to prevent podoconiosis					
Strongly disagree	39	14	1	1	
Agree	50	12	1.4 (0.6, 3.6)	4 (1.03, 16.4)	0.001
Strongly agree	47	26	0.6 (0.29, 1.4)	5 (1.30, 21.3)	0.001
Wealth index quintile					
Highest	15	1	1	1	
Fourth	38	8	0.3 (0.04, 2.75)	0.5 (0.0.03, 7.6)	NS
Middle	39	6	0.4 (0.05, 3.9)	0.7 (0.04, 10.8)	NS
Second	26	13	0.13 (0.02, 1.1.2)	0.14 (0.009, 2.2)	NS
Lowest	18	24	0.5 (0.006, 0.41)	0.04 (0.003, 0.77)	0.036

AOR: Adjusted odds ratio; COR: crude odds ratio; NS: not significant.

## Discussion

The success of podoconiosis control and elimination is contingent upon scaling up existing prevention and morbidity management services. The current prevention strategies for podoconiosis consist of regular footwear use, foot hygiene and covering house floors. Previous studies have indicated that there are significant numbers of barefoot individuals in the study area.^4,22^ Understanding how willing the communities are to buy footwear is important for program planners and district managers.

Overall, almost three-quarters (314, 74.8%) of respondents were willing to pay for footwear. Slightly fewer people with podoconiosis than without (136, 72.3% vs 178, 76.7%, p=0.30) were willing to pay for footwear, but this difference was not statistically significant. The median amount that people with and without podoconiosis were willing to pay for footwear was 100 and 120 ETB (approximately US$5 and US$6), respectively. Of those who were willing to pay for footwear, one quarter (35, 25.7%) of people with podoconiosis and nearly one-third (58, 32.6%) of people without were willing to pay the full cost of leather footwear; the corresponding figures for canvas shoes were 40 (30.7%) and 63 (35.4%) respectively. The majority of people with and without podoconiosis (118, 62.8% and 172, 74.1%) were willing to pay for shoes for their families

The overall proportion of people willing to pay for footwear in this study was 314 (74.8%), while that among people with podoconiosis was 136 (72.3%). In a study restricted to patients, 76.2% were willing to pay at least 50% of the shoe production cost if other treatment services were provided free of charge.^10^ The proportion of respondents willing to pay for shoes decreased as the specified price per pair increased: only 2% percent of participants were willing to pay at 370 ETB (US$18.50), around 10% were willing to pay more than 250 ETB (US$12.50) and 30% were willing to pay 50 ETB (US$2.50) or less. A similar trend was seen in a previous study conducted in the study setting.^10^ The proportion of people willing to pay for shoes is lower than that willing to pay for insecticide-treated bed-nets in Arbaminch, southern Ethiopia (86.0%), and Amhara region, northern Ethiopia (93.8%).^23,24^ This may be because individuals perceive malaria to be more severe than podoconiosis and participants in the bed-net studies have a clearer understanding about the cause of malaria and how to prevent it. Almost all (96.6%) knew that malaria was caused by a mosquito bite and that bed-nets are effective for prevention.^24^ However, many people involved in the podoconiosis study held one or more misconceptions about its cause. More than half of people with and without podoconiosis said that the cause was magic. Only 12 (12.0%) and 14 (11.3%) of people with and without podoconiosis said that red clay soils caused podoconiosis. A community-based study on perceptions about the cause, prevention and control of podoconiosis conducted in northern Ethiopia showed that 41.3% did not know its cause. Only 18% of participants said that podoconiosis was caused by barefoot walking and only 37.5% believed that podoconiosis was preventable.^11^ In the earlier willingness to pay study, only 8.6% of participants mentioned that red clay soil was associated with the development of podoconiosis, and 91.4% had at least one misconception about the cause of podoconiosis.^10^

The proportion of people willing to pay for footwear in this study was higher than the 68% proportion willing to pay for injectable contraceptives in Tigray.^25^ This may be because contraceptives are less acceptable than footwear in terms of religion and culture, though cultural barriers to wearing shoes or boots to farm have been reported.^22^ The proportion of people willing to pay for footwear in this study was lower than the 93% proportion willing to pay for prevention of lymphatic filariasis in Haiti^26^ This difference might be due to differences in socio-economic and demographic characteristics of the study populations and difference in awareness (more people know the exact cause of lymphatic filariasis).

Among people with podoconiosis, 92.6% and 79.3% used footwear when walking to market and church, respectively, but the majority did not use footwear when at home or during harvesting. This pattern of use is similar to that in the study conducted in Wolaita, which showed that adults used footwear for social events and gatherings including market attendance, church services, weddings and funerals. Farmers rarely wear shoes or boots while working in the fields, and many householders did not use them while gathering wood or fetching water.^22^

The proportion of people willing to pay is slightly lower among people with podoconiosis than without. This may be because people with podoconiosis are less able to work and gain income than people without podoconiosis. This is supported by the finding that the median monthly income of people with podoconiosis is lower than that of those without podoconiosis. The perceived income of people with podoconiosis in reference to their neighbours is lower than that of people without podoconiosis. Most people with podoconiosis fell into the lower wealth index quintiles. This is in accord with an earlier study of the economic costs of podoconiosis in southern Ethiopia, which demonstrated total direct costs of US$143 per patient per year. The total productivity loss for a patient amounted to 45% of the total working days per year, causing a monetary loss equivalent to US$63.^27^

In this study, people without podoconiosis who were single were considerably less likely to be willing to pay for footwear compared to married respondents. This may relate to differences in economic empowerment by marital status. In rural communities such as those studied here, most single individuals do not own farmland. Willingness to pay was positively affected by perceived household income: it was found that people without podoconiosis who perceived that their household income was ‘average’ compared to their neighbours were more likely to be willing to pay compared with participants who perceived their income to be ‘below average’. This is in accord with findings of the study assessing willingness to pay for podoconiosis lymphoedema treatment, in which study participants who perceived their households to be poorer than the village average were willing to pay about half as much as those who considered their socioeconomic position average or better.^10^ Most of the study participants (137, 72.9% with podoconiosis and 171 (73.7%) without) perceived their household income to be ‘average’ in reference to their neighbours. According to the study on willingness to pay for podoconiosis lymphoedema treatment, the majority (64.1%) of the study participants perceived their household's socioeconomic status to be lower than the village average.^10^ The possible explanation for this may be people who perceived their household income to be ‘average’ compared to their neighbours may have been more economically mature. This study also revealed that people without podoconiosis who strongly disagreed that action should be taken to prevent podoconiosis were less willing to pay compared to those who strongly agreed. People without podoconiosis and who had the lowest economic status were less likely to be willing to pay for footwear. This is the same as the study which showed that willingness to pay for podoconiosis treatment was strongly associated with the financial status of the household.^10^ Women with podoconiosis were less likely to be willing to pay for footwear than men, once again echoing the willingness to pay for treatment result.^10^ One possible reason for this could be that in most rural communities in the study area, men take the primary responsibility over decisions about payment.^28^

People with podoconiosis who were unable to read and write were less likely to be willing to pay for footwear than those who could read and write. Similar study findings have been reported in previous studies conducted in Ethiopia on willingness to pay for insecticide treated bed nets and injectable contraceptives.^23,25^ Furthermore, people with podoconiosis in the lower quintiles of economic status were less likely to be willing to pay for footwear than those in the higher quintiles. Studies have shown that financial limitations are the primary barriers against consistent use of footwear among people with podoconiosis.^22,27,29^

This study has several limitations. It was conducted in woredas in which IOCC interventions exist, possibly leading to an overestimation of willingness to pay. Some important variables were not included in this study, such as size of shoes. This study focuses on self-reported willingness to pay which is not identical to ability to pay. Finally, social desirability bias might lead to overestimation of willingness to pay.

### Conclusions

There is substantial willingness to pay for footwear in northern Ethiopia, where podoconiosis and many other NTDs are highly prevalent. The willingness to pay expressed here indicates potential demand for footwear in the community. This may be of interest to shoe companies. The promotion of self-financing could enhance the sustainability of shoe wearing. The proportion of people willing to pay is slightly lower among podoconiosis patients, but this is not statistically significant. Willingness to pay studies help identify factors that, if addressed through social mobilization, subsidies, or other strategies, may improve footwear use rates, which is critical to the elimination of podoconiosis. The proportion of people who are willing to pay for footwear to prevent disease has important implications for the ability of the control program to achieve reduction of new cases of podoconiosis. This study focuses on willingness to pay and did not address the financial capacity of the respondents to pay for footwear. Therefore it is difficult to measure the actual demand of the community; hence further research on the financial capabilities and the actual demand of the community for footwear is warranted.
